# Why and How to Prefer a Causal Account of Parenthood

**DOI:** 10.1111/josp.12059

**Published:** 2014-06-02

**Authors:** Lindsey Porter

**Affiliations:** Lancaster University

## 1. Introduction

The most promising account of parental obligation currently on offer is a causal account. According to such an account, parents incur obligation to their offspring by causing them to exist. Causal accounts are explanatorily strong—the moral force of causing someone to exist seems straightforward. They seem to more or less capture our everyday intuitions about who is a parent with parental obligation and why. However, causal accounts are not problem-free. Importantly, causal accounts do not pick out adoptive parents as parents with parental obligation. Further, causal accounts *do* seem to pick out gamete donors as parents with parental obligation. The difficulty in fitting nongenetic parents into such an account—as well as that of excluding nonparental progenitors (like gamete donors)—has led some to reject the causal account in favor of a consent, or “voluntarist” account of parental obligation.

Voluntarist accounts, like causal accounts, seem to have a clear moral mechanism: where obligation is acquired by causing existence on a causal account, it is acquired by consent or volunteer on a voluntarist account.^1^ Indeed, a voluntarist account of parental obligation is squarely in line with standard philosophical thinking about role obligations more generally: we have role obligations in virtue of having consented to take on the role to which the obligation attaches.^2^ However, this mechanism seems to fly in the face of our basic assumptions about the nature of parenthood and parental obligation. Importantly, we tend to think that parents are obliged to foster the well-being of their children even if they do not want to; that is, even if they do not consent to do so. We think that parents, even and perhaps especially unwilling parents, can and ought to be held to account with regard to their obligation to their children.^3^

So, we seem to be left with two equally strong, but equally flawed accounts of parental obligation: a causal account, on the one hand—one that seems to “get it right” with respect to the nonvoluntariness of parental obligation, but wrong on who has such obligation—and a voluntarist account, which seems to match intuitions about nonbiological parents and nonparental progenitors, but seems to inappropriately conceptualize parental obligation as optional.^4^

Elizabeth Brake has argued that a voluntarist account of parental obligation should be preferred over a causal account of parenthood.^5^ Causal accounts, Brake argues, suffer (at least) two conceptual weaknesses: there is an explanatory gap between being the metaphysical cause of someone's existence and owing that someone care; and causal accounts belie the socially constructed nature of parenthood itself. Given these weaknesses, Brake argues that the voluntarist account is the stronger of the two accounts.

I will argue that the explanatory gap between causing and being obliged can be filled on a more plausible conception of the nature of the obligation; and that, while a simple causal account cannot answer Brake's concerns about the scope and nature of parenthood, there are easy and intuitive modifications available to a causal account, such that a modified causal account can be consistent with recognition of social construction.

In section 2, I present Brake's arguments against a causal account. In section 3, I present her positive account and give some reasons for thinking we ought not bite the voluntarist bullet. In section 4, I argue that a causal account that recognizes the difference between being a (social) parent and being a progenitor (what I will call a “maker”) is not vulnerable to Brake's social-construction objection. Finally, in section 5, I present an alternative to Brake's libellous-causing account of the moral power of causing: a broadly Kantian account of causing existence as choosing for.

## 2. Why Brake Wants Volunteers

On the face of it, causal accounts of parental obligation have much to recommend them. According to a causal account, parents are obliged to care for their children in virtue of having caused their children to exist. On this picture, then, we have an apparently coherent account of why parents are obliged that fits with other accounts of obligation: parental obligation is a sort of liability; it is acquired in virtue of the morally weighty action (causing existence) of the parents.

Causal accounts, importantly, have teeth. One does not have to be willing to be held to account. The parent who says “I don't want to” has failed, on a causal account, in her moral duty.^6^ Consent accounts, on the other hand, appear to be unable to hold “deadbeat” parents, feckless neglecters, and the like, to account. But Brake argues that causal accounts have (at least) two serious problems, and as such we ought to reject them. In section 4, I will argue that these worries arise for a causal account only if we fail to notice the ambiguity in the term “parent”; and that by picking apart the (at least) two distinct roles salient to discussion of parental obligation, we can dissolve Brake's worries. First, I will rehearse the worries below.

### 2.1 Libellous Causing

Brake's first worry about causal accounts is that the connection between causing and being obliged is not so clear as it may first appear. One worry about causing as an obligation-motivator is that it is unclear who should count as a cause: are obstetricians causes? Are gamete donors? Are the parents' matchmakers? A causal account of parental obligation needs a solid account of causation, on pain of picking out the wrong people as parents, and the account must be noncircular. In section 3, I will discuss this smaller worry further and argue that an appropriate conceptualization of causation is available to the causal theorist. However, a larger worry, according to Brake, is with the very conceptualization of parental obligations as compensatory obligations: how do we get from causing human existence to owing care?

Brake begins by pointing out that only some acts of wronging an individual oblige us to compensate them for that harm. For example, if I hurt your feelings, I am presumably not obliged to compensate you for this hurt. However, if I damage your car, I probably am. So, an account is needed of why causing someone to exist is the sort of harm that generates compensatory obligations. Brake argues that such an account is unavailable.

The first step in explaining the link between causing existence and being obliged to compensate is to motivate the claim that causing someone to exist results in the right sort of harm. Brake notes that compensatory obligations are rectificatory. They are obligations to fix bad states of affairs for which one is morally responsible; to “make the victim whole again.” The harm that generates them, then, must be either a rights violation, or a “serious boundary-crossing harm” (2010, 159). So, we will need an account of why (or in what sense) causing someone to exist is either a rights violation or a serious boundary-crossing harm.

Some theorists have supposed that the child's neediness is the relevant harm. Making someone needy is, plausibly, harming them in a way that generates a compensatory obligation. When one causes a child to exist, one causes an individual to be needy since babies and children just are needy. The compensatory obligation, then, is the obligation to rectify the child's neediness. Causing neediness, then, would count as a serious boundary-crossing harm. Alternately, we might suppose that causing existence generates rights that must not be violated. Brake considers Feinberg's claim that children have a “right to a reasonable assurance of a minimally decent life” (Brake 2010, 159). On this account, causing a child to exist would be a rights violation in the absence of care.

For the sake of argument, Brake assumes that both of these claims are correct: that causing neediness is a harm that demands compensation; and that failure to provide reasonable assurance of a minimally decent life would constitute a rights violation. The harm and the potential rights violation together, then, would generate what Brake calls “procreative costs”: what one owes to the child in virtue of the harm done and of the child's rights.

On this picture, one would have compensatory obligations toward one's offspring in virtue of having caused the child to exist. However, Brake points out, parental obligation far outstrips procreative costs. If neediness is the bad state of affairs that must be rectified, then the compensation ought to consist of making the child nonneedy. Clearly, children cannot be made non-needy; at best, their neediness can only be minimized. Minimizing neediness, it seems, would require the parent to enable the child to meet her own needs as far as is possible. If a reasonable assurance of a minimally decent life is the right that must not be violated, then the obligation is just to provide reasonable assurance of a minimally decent life.

But parents surely owe their children more (and different) than simply enabling of self-sufficiency and opportunities for minimal decency. Importantly, we think that parents owe their children love, interpersonal warmth, and close involvement in their lives for at least many years. So, even if it is right to suppose that children are harmed by being brought into existence, and that children are put in danger of having their rights violated by being brought into existence, it seems that the compensatory obligations generated by this harm and this right cannot fully describe parental obligation.

Furthermore, the extent to which parents must provide for the well-being of their children is significantly dependent upon social circumstances. A child raised in a society that lacks basic sanitation, educational opportunities, and so on is by default further off the “minimally decent” mark than is a child raised in a society that offers robust social care. This means that what parents are obliged to, on this compensatory model, will vary with social circumstance. In this sense, the child's “neediness” is not caused by the causal parents alone.

So there is a gap. I will argue that this gap is only apparent and that compensation is simply not the appropriate model for understanding parental obligation. In section 4, I will argue that a rectificatory account of the connection between causing existence and owing care is not the appropriate account of the moral weight of causing existence. I will argue instead that we ought to understand causing existence as a (unique) instance of choosing on another's behalf and the obligation it generates as an obligation to choose well.

### 2.2 Parenthood as a Social Construct

Brake's weightiest worry about the causal account is a worry over the social or institutional nature of parenthood. Who we take to be parents depends upon how our societies are structured. Likewise, what parental obligation consists in is, in Brake's words, “institutional, not natural”: both what children need, and what parents are expected to provide them with, are delineated by our social practices. Parenthood, then, is socially constructed. This seems to pose two different sorts of problems for a causal account. First, it seems like it simply cannot be the case that certain people (e.g., biological progenitors) belong to the category “parent” of necessity, or “naturally.” And second, it seems that it cannot be the case that parents, of necessity, have the obligations we intuitively assign to parents.

Parental obligations are role obligations; that is, they are obligations one has only in virtue of being a parent. So, parental obligations will apply to anyone who occupies the role. It is a challenge for any theory of parenthood to get this right: any good theory needs to explain why *parents*—that is, the people we (pretheoretically) pick out as parents—are obliged.

As a point of clarification, Brake differentiates among three sorts of parenthood: moral parenthood, social parenthood, and legal parenthood. Legal parents are just those who the law holds accountable for the care of children. Social parents are those who fill the social, care role of parent for the child. Moral parents are those who we take to be morally responsible for the care of the child. These three sorts of parenthood come apart. For example, an absent father may be a legal parent—obliged by law to pay child support, say—while having never met a child and thus not fulfilling the social role of parent. Grandparents who take on the full-time care of their grandchildren might be social parents, although we might not think them morally obliged to provide such care. And so on.

The primary concern of a (moral) theory of parenthood is to fix moral parenthood; that is, to assign obligations to all and only those people who are moral parents. This task is not straightforward. For example, a simple biological account of parenthood will not pick out all and only those we take to be parents, since we usually think that gamete donors are not parents, despite being biological progenitors. A simple causal account will not do, since adoptive parents—who we take to be parents—do not cause their children to exist.^7^ So, properly delineating the role of parent is a challenge for any theory of parenthood.

Brake's worry, however, goes one step further: the worry is that there is no hard-and-fast delineation of parenthood, or rather, that because “parent” varies so greatly in its meaning across history and cultures, we must conclude that there is no objective moral category to be delineated. It seems like parents are just *those who parent*. If this is right, then it is difficult to see how we can get from a natural fact—like biology, or causation—to the moral fact of parenthood.

One might be tempted to reply as follows: “even if we use ‘parent’ in many ways, still we can talk about *parents*; that is, even if someone, say, acts *as a parent*, and we call him ‘a parent,’ still we can separate him, in our theory, from a *real* parent. And it's the real parents who have the obligations.”

But who might these real parents be? We cannot say that the source of the sperm and the source of the ovum are the “real” parents since gamete donors are not parents. We cannot say that either the biological parents or the adoptive parents are the parents, since adoption just happens to be the legal convention by which we in the west in this day and age formalize familial arrangements centered around children, when we do formalize them. In some matrilineal societies, Brake points out, children are traditionally raised by their mothers and their mothers' families—for example, by mother and maternal uncle. In most societies until relatively recently, children were cared for by larger extended families. Nuclear mother/father families are our paradigm setting for childrearing by social convention, and social convention surely cannot drive moral fact.

One might be tempted still to say that, while the extended family may have cared for the child, still the extended family is not *the parents*. But it is not clear how we can make sense of this stance. In the case of matrilineal communities, for example, where mother and maternal uncle traditionally raise a child, the male progenitor is in the same relationship to the child as is a sperm donor in twenty-first century Western society. If the sperm donor is not a parent, it seems unlikely the male progenitor in the matrilineal society is. If the nonbiological male social parent in cases of sperm donation is the father (and surely he is), then it seems like the mother's brother is the father in matrilineal societies. And there are examples much closer to home: many grandparents, for example, raise their grandchildren. It does not do to point to the fact that the children do not call them “parents”: children in China do not call their parents “parents” either, and yet they are.

Fixing parenthood seems a serious difficulty for a causal account of parenthood.^8^ On the one hand, if we understand causation narrowly—such that, for example, only those immediately responsible for conceiving and gestating the embryo that becomes the child are taken to be causes of the child's existence—then our conception of parenthood is tethered to our narrow social understanding. As Brake puts it, it would seem simply to “reflect the heterosexual nuclear-family practices common in contemporary western society,” rather than to rightly pick out moral parents (2010, 167).

On the other hand, if we understand causation in a way that takes social practice into account, then the moral force of causing seems to melt away. For example, we might say that maternal uncles in matrilineal societies are causes of a child's existence: the child is conceived, gestated, and birthed on the understanding that the uncle will be a primary carer to the child, and has responsibility for the child's wellbeing. The social understanding that he will fulfil this role is part of the circumstances in which the child is brought into being, and he has actively played this role. Therefore, he is a cause.

But if we understand cause in this way, causal parenthood becomes impossibly large and indefinite, and the causal account loses its teeth. Fixing moral parenthood will depend on contingent social facts, rather than anything metaphysical; most plausibly, it will also depend on the intentions of prospective moral parents. This will mean, in effect, that one is a cause of a child's existence only if she intends to parent. Causation will drop out entirely in this case, and the account will collapse into voluntarism. Parents will be those who choose to be so, and causing will lose its explanatory power altogether. Brake, then, concludes that the causal account ought to be abandoned and a straightforwardly voluntarist account given.

## 3. Brake's Strong Voluntarist Account

Because causal accounts seem to fall down with respect to motivating compensatory obligation, and fixing moral parenthood, Brake argues that a voluntarist account of parenthood is, after all, the most plausible account we can give. In Brake's terminology, moral parenthood springs from social parenthood, and whether one is a social parent depends upon whether one has consented to occupy the social role parent.

### 3.1 Strong Voluntarism

On standard consent accounts of parenthood, consent to parent is sufficient, but not necessary for parental obligation.^9^ This sort of account makes sense of the obligations adoptive parents, and *de facto* (nonbiological) parents are under with respect to their children, but leaves open the question of how such obligation can be otherwise acquired. Brake defends a stronger version of the consent account, on which consent is necessary, but not sufficient for parental obligation. So on this account, one acquires parental obligation by consenting—either explicitly or tacitly—to parent and by being in the appropriate rights position with respect to the child.^10^

The plausibility of this view, Brake writes, “derives from the thought that obligations limit liberty, and liberty can only be limited through choice or as a result of wrong-doing” (2010, 156). Because, as we have seen, conceiving of parental obligation as compensatory obligation fails, the curtailment of liberty that parental obligation constitutes must be voluntary. Brake considers the objection that many special obligations—for example, filial obligations—seem to be nonvoluntary: it seems as though we have duties to family members that we did not choose. In that case, it is unclear why we should need to *choose* to have parental obligations; why parental obligations should not be like other sorts of familial duties. She suggests that these nonvoluntary obligations may be “duties of virtue”—rather than duties of justice—that do not correlate to moral rights (2010, 156). In other words, while I may have filial duties toward my parents, even though I have never consented to have such duties, my parents do not have a right that those duties be fulfilled. I fulfill my duties toward my family as a matter of virtue. If I do not, I am perhaps vicious, but I have not violated anyone's rights. In contrast, if I fail in my parental obligations, I have exactly violated my child's rights; I have perpetrated an injustice against my child.

### 3.2 Broad Consent

That parental obligation requires consent does not, at first blush, square with the realities of becoming a parent. Many parents become so either under pressure from family, partners, society, and so on; or simply accidentally, or even fecklessly, carried along by fate. One may even become a parent through need arising by chance—due to the sudden death of a child's birth parents, for example. The notion that parents usually become so by careful, reasoned, intentional action is implausible. Brake acknowledges this, and employs a broad understanding of consent, on which it might be careful and intentional; or it might simply be “tacit voluntary acceptance” of the role—one may choose it, actively, or one may simply not decline to enter into it (2010, 157). Echoing Thomson,^11^ she writes that

… once someone has chosen not to abort, undergone prenatal medical care, bought some baby clothes, and taken an infant home, the role of parent has been tacitly accepted. In our society, taking a child home as one's own counts as assuming the role of parent—there is no other way to describe this activity, except as baby-snatching. If abortion is an option, then choosing to continue a pregnancy without making plans for adoption constitutes accepting the role of parent.^12^

In other words, one typically consents to parent by doing it, by parenting. And once one has done so—once one has taken up the social role—one is thereby morally obliged. There is no other way, according to Brake's account, to acquire parental obligation. All parental obligation is (broadly) voluntary.

### 3.3 Deadbeat Dads

Voluntarist accounts are vulnerable to an objection that Brake has dubbed the “deadbeat dads” objection. Indeed, this objection seems to be the strongest objection to a voluntarist account. Brake argues that the worry is only apparent: that the intuitions we have about “deadbeat dads” can be adequately handled by broader moral considerations in a way consistent with the voluntarist account.

According to the deadbeat dads objection, a voluntarist account is implausible because so-called deadbeat dads cannot be held to account: fathers who abandon their children and fail to provide support cannot be said to have violated their child's rights nor ought they be subject to moral scrutiny for having declined to support them. Voluntarist accounts seem to be unable to attribute blameworthiness in the face of global parental failing.

Brake argues that the intuition that biological fathers who abandon their children are blameworthy is not apt as a criticism of the voluntarist account. To the extent that “deadbeat dads” ought to be held to account, she argues, they are not so obliged on parental grounds. Thus, although the voluntarist account cannot, on its own, explain why abandoning one's offspring is morally reprehensible—or why fathers ought, for example, to be obliged to pay child support—this does not tell against the account because we can explain these judgments in other ways.

First, Brake writes, fathers “owe general duties of rescue to the infant” (2010, 174). Just like everyone else, fathers have a general duty not to knowingly abandon a child to its death. Arguably, biological parents are in a particularly good position to aid their biological children. And anyone in a good position to save a helpless child from imminent death or danger has a (general) duty to do so. So, since biological fathers are often uniquely situated to rescue their children, they have a general (nonparental) duty to do so if need arises.

Second, there are strong social-justice reasons in favor of assigning legal parenthood to biological fathers, even if we cannot assign moral parenthood to them. Most notably, single mothers and their children are among the most vulnerable in our society. Assigning legal support duties to biological fathers is in keeping, then, with maintaining social justice by alleviating the vulnerability of both the child and the mother.

And finally, Brake argues that “‘deadbeat dads’ usually describes men who accept obligations and then abandon their families” (2010, 175)—social (and thus moral) dads who walk out on their children. In these cases, a voluntarist account is perfectly able to attribute wrongdoing since these “deadbeat dads” are parents with parental obligation on a voluntarist account.

On the other hand, some people we might want to describe as “deadbeat dads” are not social parents who have abandoned their children. They are what Brake calls “reckless procreators”: men who do not take precaution against procreation, but do not subsequently take up the parenthood role. These deadbeat dads cannot be held to account *qua parents* on a voluntarist account, but the blameworthiness of their behavior can be otherwise explained. This variety of deadbeat dad, according to Brake, has harmed the mother: “Pregnancy has life-altering, sometimes devastating, social, economic, and physical effects on women, and reckless male procreators may be rightly blamed for subjecting them to these.”^13^ So, while reckless procreators have not failed in any parental obligation—since they have none—still they can be rightly criticized for harming the mothers of their children on Brake's account.^14^

But, while this reply to the deadbeat dads worry can account for blameworthiness, it does not get the right sort of blame in. What seems particularly blameworthy about the deadbeat dad's behavior is that he has harmed *the child*, not the child's mother—although of course, he has probably done that also. This is clear when we consider a situation in which the birth father has declined to provide care for the child and the mother is unable adequately to do so on her own. She is probably harmed, but what troubles us particularly is that the child is harmed: we feel the child is harmed by the birth father, not (simply) the mother.^15^ And the intuition is that he owes the child (at least) rescue because he bears a unique relation to the child and not simply because he is like anyone able to rescue.

To describe the deadbeat dad as violating a general duty of rescue does not seem to capture the intuition about what is wrong with the abandonment.^16^ After all, doctors and nurses in hospital are probably in a *better* position to provide rescue to the baby in the first instance, but we do not have the intuition that they must provide sustained care or support.^17^ An account that could preserve the intuition that the birth father, in particular, owes the child care ought to be preferred.

Furthermore, in the absence of a motivation for picking out the biological father as being in a particular moral relation to the child, it is unclear why it would serve justice to assign legal parenthood to him, even given what justice demands for the mother. Why not tax all males of sexual maturity? Why not assign legal parenthood to the mother's nearest male kin—brother or father, say? It seems unlikely that a purely practical reason for holding the father to account could justify compelling him in particular to provide care if we cannot attribute moral parenthood to him. The biological father seems an intuitive choice for legal parenthood precisely because he, unlike other men of sexual maturity, is the reason there is this particular child who needs a legal parent. In other words, assigning legal parenthood to the biological father makes sense because he stands in an appropriate moral relation to the child.

Finally, the claim that proper deadbeat dads—that is, those who “stick around” and then abandon—are blameworthy, whereas “feckless procreators” are not strains the concept of tacit consent beyond usefulness. In many cases, these fathers do not, as Brake describes, buy baby clothes or take the baby home. Indeed, these fathers may in many cases play no more of an active role in the child's early life than do the grandparents or the mother's friends. If we are to say that a man who is the biological father of a child has consented to father the child in virtue of having “stuck around,” then it seems either that the mother's friends have also consented to be the father of the child—and are thus also fathers of the child^18^ —or that the biological (or causal) relationship is necessary for the father to be a father. But if this is so, the consent account will quite obviously lose the plausibility edge it seemed to have over the causal account.

The problem with Brake's reply to the deadbeat dads objection is just that voluntarism about parenthood is implausible. More broadly, the idea that morality cannot curtail our liberty without our consent is implausible. The relationships we find ourselves in with other persons—whether we enter them purposively and voluntarily (as with friendships or promises), or simply find ourselves in them (as in familial relationships, or even as bystanders or neighbors or fellow citizens)—plainly do generate moral obligations. We do have special obligations that we do not choose, and this is borne out in a great many of our everyday moral intuitions.^19^

However, Brake is right that an insistence on the deadbeat dad's having violated a special obligation to the child is question-begging in the absence of a plausible alternative account of such special obligations. After all, feckless procreators, anyway, are clearly not social parents. In the next section, I will present a causal account of parental obligation—drawing on the work of David Archard—and show how such an account avoids Brake's social-construction objection, and in the final section of the paper, I will present an alternative to the “liability” model of causal parenthood: a Kantian conceptualization of causing to exist as morally meaningful “choosing for.”

## 4. A Bifurcated Causal Account of Parenthood

In this section, I will present my positive account of parental obligation—what I am calling a “bifurcated causal account”—according to which causing a child to come into existence places one in a distinct moral role to which obligation attaches, but does not make one a *parent*; while occupying the role “parent” also obliges one to one's child(ren). I will call the role one enters when one causes a child to exist the “maker” role, and the role one enters upon becoming a (social) parent simply “parent.” I will argue that being a maker implies a *pro tanto* (but defeasible) duty to take on the role parent, but also implies obligations to the child even if the role “parent” is not taken up.

A straightforward causal account of parenthood, unlike the voluntarist account, meets the challenge of the deadbeat dads objection. On a causal account, those who bring children into existence, by procreating or instigating procreation, are obliged to the resulting child because the child's existence and existence-with-rights is a result of their actions.^20^ It is more flexible than a biological account since nongenetic procreators can still be causes. However, a simple causal account is not without difficulties. As Brake and others have pointed out, it is hard to make sense of a causal account in the face of the social-construction of parenthood.^21^ Social parenthood and moral parenthood do not match up on a simple causal account in nonhetero/nuclear parenting cultures and families. Further, it is difficult to know what to say about gamete donors (among others) on a causal account. It seems like gamete donors are causes, but we tend to think that gamete donors are not parents. And if we include nongenetic procreators^22^ as causes, it is difficult to exclude others involved in the conception—IVF technicians, for example. (They are a particular worry when donated gametes are used since the technicians are often the ones who actually conceive the embryo.) However, contra Brake, these worries need not doom the causal account: they simply show that subtlety is needed.

Causal accounts are problematic in the context of nonhetero-nuclear-biological families because the concepts usually employed in discussion of parenthood are hetero-nuclear-bio-centric. In particular, the very language we use in the everyday to discuss parenthood obfuscates the distinction between those who cause children to exist and those who are primary carers of children: both, in everyday language, are called “parents.” It is no surprise, then, that what we say about “parenthood” does not gel with our strong intuitions about nonbiological parents. What is needed to rescue the causal account is simple conceptual clarity. Causal accounts are highly successful accounts of what obligation one incurs in virtue of causing a child to exist, but causing existence and being a parent need not go together. A causal account that recognizes this can meet the challenges Brake sets.

Parenthood—of either sort—is a moral role. When one occupies the role of parent, one has obligations in virtue of being in that role. The obligations attach to the role, in the first instance, and in some sense only derivatively to the person in the role. That is, for any given person in a moral role, they do not have the role obligations by necessity, or in virtue of being a member of the moral community, and so on. Rather, they have the obligations only in virtue of occupying that role.

Parenthood, as with all moral roles, has not only obligations attached to it, but also has entrance and exit conditions. When Brake claims that parenthood is voluntary, her claim is just that the entrance condition on the role “parent” is consent. When the standard causal theorist claims that parenthood is causal, her claim is just that the entrance condition on parenthood is *causing the child to exist*. But of course, not all those people we call “parents” cause their children to exist, and not all those people who cause children to exist count as “parents” on standard linguistic usage. One way of responding to this apparent problem with the causal claim is to say that causation must not be the entrance condition on parenthood. The other—the one I advocate—is to say that there are two distinct moral roles in play when we discuss social parents, on the one hand, and progenitors or causers, on the other, each with different entrance and exit conditions, as well as different obligations attached to them.

On this sort of causal account, then, both the role of *parent* and that of *maker*—where “maker” includes both progenitors, and other causers like intended parents who use donated gametes, as well as (potentially) others—are morally weighty roles, and both imply obligation to the child in question. Maker obligation, then, is obligation incurred in virtue of having caused the child to exist (entrance condition) and is, roughly, the obligation to make the child's existence a good one to the extent that one can. Parental obligation, on the other hand, is the obligation incurred in virtue of taking on the social role of parent (entrance condition) and includes all those every day care obligations we would normally attribute to the social parent. This account implies a pluralist conception of entrance conditions on the role *parent*. But we can say more than that: in addition to consent—the means by which adoptive parents, for example, enter the role *parent*—one might also enter into the role of parent because one is obliged to do so, and one is obliged, *pro tanto*, to enter into the role of parent by occupying the role maker.^23^

David Archard (2010) differentiates between these two moral roles by drawing a distinction between what he calls parental *obligation*, on the one hand, and what he calls parental *responsibility*, on the other. This distinction, on his account, picks out the difference between “the obligation to ensure *that* someone acts as a parent to the child” and “the responsibilities of *acting as a parent*” (104). Causing a child to exist, on Archard's terminology, generates parental obligation: the duty to the child, to ensure it has proper care. Being a parent generates parental responsibility: the duty to actively care for the child. Archard writes that

… *if* someone *does* incur a parental obligation to make provision for a child they have caused to exist then he or she is not under a duty to provide that care themselves … a simple causal theory of parental obligation is not defensible if it holds that causing a child to exist is sufficient to incur [parental responsibility]. (114)^24^

So, on Archard's account, causing a child to exist does not make one a parent, and it does not generate parental responsibility—duties to actively care for the child. What it does is generate a duty to ensure that the child is well parented, and this duty can be discharged either by parenting, or by securing parents for the child other than oneself.

This distinction gets around Brake's worry about the social construction of parenthood, in that it does not misattribute parental *responsibility* (what I am calling *parental obligation*, in contrast to *maker obligation*) to nonparental progenitors. But one might worry that it does so at the expense of the larger strength of a causal account. A big reason to prefer a causal account, again, is that it has teeth: it can hold negligent parents to account. But if parental obligation is too weak, and if the connection between obligation and responsibility is too tenuous, it seems the causal account may lose those teeth.

The worry might go as follows. It seems like we want to say that children have a *right* to be cared for. And it seems that, in order for saying so to mean anything, we need to say that children have this right *against someone*. That is, there needs to be some particular person or persons from whom the child has a right to care.^25^ If the causal account can provide no such person, then its plausibility is significantly diminished.

We might want to say that having a duty to see that someone cares for the child can adequately motivate a rights claim on behalf of the child. We might say that the child's right is that she be parented, and the causer's obligation is to make it the case that she be parented. But on inspection, it is not so simple as this. In the usual run of things, the child's right to be cared for or parented is met, and there is no worry over who the right is against. But one must simply imagine a situation in which *no one*, including the causal parent, wants to parent a given child in order to see the problem.

If the birth parent tries his best to find a parent for the child but, because no one (including himself) is willing to volunteer, fails to find one, the causal parent seems to have fulfilled his parental *obligation* (on Archard's terminology)—or anyway, the child has no strong claim against him.^26^ The causal parent has tried his best; no one is willing; and since the causal parent has obligation but not responsibility, there is so far no reason to say that the causal parent, more so than anyone else, ought to care for the child if unwilling. And yet, the child's right to be cared for will have been violated. So, there is an asymmetry between the causal parent's obligation and the child's right. That is, the child's right cannot be paired with the causal parent's obligation. The right cannot be against the birth parent on this account. It is unclear, then, against whom we can say the child has a claim when that right is not met.

We might think to say that the child's right is against the (social) parent if and when one is acquired. But then, in a case where no willing parent is found, we would be forced to say that the child has no right to be cared for. Surely, if any child has a right to be cared for, all children do, regardless of who is keen to parent them. So, if parental *obligation* is simply the duty to make sure someone cares for the child, and does not imply a duty to do the caring, we will have to say that children do not have a right to be cared for. And this seems wrong.

In order for a modified causal account to retain the plausibility and theoretical strength that makes a causal account attractive in the first place, the connection between *obligation* and *responsibility* needs to be stronger—that is, the obligation one incurs in virtue of filling the moral role *maker* must include some sort of obligation to take up the role *parent*.

My claim, then, is that causing a child to exist generates a *pro tanto* obligation to enter the moral role of parent. The obligation that attaches to the role maker, then, will be to make it the case that the child is cared for (or more broadly, to make the child content with her condition, in so far as one is able), and this will imply a *pro tanto* duty to do the caring oneself. So, on my account, parental *obligation* and *responsibility* (in Archard's terminology) are intimately morally related. (Causal) parental *obligation*, then, is in part an obligation to enter into the role that generates (social) parental *responsibility*.

On this sort of causal account, the child's primary right to be cared for will be against those people who fill the moral role *maker*; that is, those who caused the child to exist.^27^ If the right is met by others—if others take up the role of parent—then the right is met. If no other parents are available to the child, the makers are obliged to parent. However, if the right is not met—if the child is not adequately cared for by anyone—then even if the makers and the parents are different people, the child will still have a claim against the makers since regardless of what duties the parents have in virtue of taking up the role, the child has a right against the makers.

At first blush, this account seems to throw up unintuitive consequences in cases of artificial conception and especially gamete donation. It might seem that gamete donors, especially, incur parental responsibility on this account since gamete donors are obviously part of the cause of the child's coming into existence. And since gamete donors are categorically not parents, this might seem like a strike against the account. But again, it is important to point out that *maker* and *parent* are distinct moral roles. Those who cause a child to exist are not thereby *parents*. But they are *makers*. The child created with their gametes has a right against them. Although gamete donors, like all other makers, will have a *pro tanto* obligation to parent the child in virtue of having caused the child to exist, this obligation will be, under normal circumstance of gamete donation, outdone by the rights and commitments of the intended parents (the gamete recipients).

That the recipients of the donated gametes have instigated the creation of the child with the express intention of parenting it, coupled with the maker obligation of those recipients, will mean that the gamete recipients are more strongly obliged (much more strongly, one suspects) to parent the child. However, it will still be the case that if the child who is created is not cared for, and her rights are violated, then the gamete donor's having brought about this state of affairs will be a rights violation: she or he will have failed in his/her obligation to the child. Gamete donors, then, will not count as *parents* on this account; but they will, along with the gamete recipients (and indeed perhaps the IVF technicians, and so on who have conceived the embryo), count as makers and will have maker obligation.^28^

If we conceptualize the obligations of makers and parents as attaching to two distinct but connected moral roles, we both dissolve Brake's worry over the social constructedness of parenthood, and retain both the moral-progressive strength and intuitive plausibility of the causal account. We can say that causing generates an obligation to the child *and* that children have a right to be parented *and* that not all causers are parents. Because maker obligation implies a *pro tanto* obligation to take on the role of parent, simply doing one's best to find a parent for the child will not always be sufficient to fulfill one's maker obligation. Maker obligation, then, will include something like a defeasible meta-obligation to care for the child. However, because this account makes clear the moral distinction between maker and parent, saying that gamete donors, for example, are obliged to the children they help create does not amount to the implausible claim that gamete donors are parents. It therefore speaks to the intuitions that both gamete donors are not parents and that morality ranges over gamete donation.^29^

## 5. Causing as Choosing For

I have argued that a modified causal account of parental obligation, on which “makers” have a duty to ensure the care of the child they cause to exist, and a *pro tanto* obligation to parent the child, can meet the challenge set by Brake's social-construction objection to causal accounts, while still retaining the core attractiveness of a causal account. However, there remains a worry over how causing someone to exist can generate care obligations toward them.

Brake's claim is that parental obligation cannot be construed as rectificatory, and thus the moral link between causing and being obliged is mysterious. In this section, I will argue that the rectificatory conception of causal obligation is not the appropriate way to conceive of the connection between causing and being obliged. Rather, the obligation should be understood as moral constraints on making choices on others' behalf.

Kant's causal account of parental obligation seems to rest on just such an understanding of causal obligation. In the *Metaphysics of Morals*, he writes that

… it is a quite correct and even necessary idea to regard the act of procreation as one by which we have brought a person into the world without his consent and on our own initiative, for which deed the parents incur an obligation to make the child content with his condition so far as they can.^30^

What is key, then, is not that one has caused harm, or that one has caused the potential for harm; but rather, that one has acted without the child's consent. The makers of the child choose, for the child, that it exists. And this obliges them to make existence, as best they can, a good choice.

In order to flesh out this choosing-for conceptualization of the moral import of causing, imagine that my friend Susan and I are dining in a restaurant, in advance of an important train journey. We have very little time and will need to order straight away if we are to eat before it is time to go. While we wait for the waiter to arrive to take our order, Susan receives an urgent telephone call. She excuses herself from the table to take the call. Predictably, the waiter appears to take our order just after Susan has gone. Since we are in a hurry, such that Susan will otherwise have no chance of eating, I decide to order a meal for Susan. She has not told me what she would like to eat. However, I do my best to make a good guess at what she would like.

When I order for Susan, I am choosing for her. In some sense, I am taking the decision away from her; but in the circumstances, this is probably not the right way to think of what I am doing. I am not acting paternalistically. I am not making the judgment that Susan is constitutionally unfit to choose for herself. I am not choosing for Susan when I know that she would rather choose for herself. There simply is no opportunity for Susan to make the choice herself. So, it seems likely that my choosing for her is permissible.

However, it is important to notice that *how* I choose for her will make the difference to whether my choosing for her is permissible. For example, if I know that Susan is allergic to shellfish, ordering the lobster is impermissible; if I know that she is a vegetarian, ordering the *foie gras* is impermissible. My choosing for Susan is permissible *if I do it right*. That is, if I do my best to choose well for her. More generally, choosing for is permissible only if the chooser does her best to choose well.^31^

In most cases, choosing for others when one has not been asked is at best not very nice and at worst impermissibly paternalistic. For example, if Susan were sitting at the table when the waiter arrived, and I decided to choose for her despite her wishes, this would be wrong. Likewise, if Susan had simply walked a few feet away from the table to admire the artwork on the wall, choosing for her, rather than calling her back to the table to order for herself would be impermissible. But given that there is no opportunity for Susan to choose for herself, and given that she will not otherwise eat, *choosing well* for her—or anyway, doing my best to choose well for her—is permissible.

Causing existence is an instance of (potentially morally permissible) choosing for. When I make it the case that someone comes into existence, I am choosing for them that they exist. Because there is no opportunity for a nonexistent person to make her own choice, we can assume that this choosing for is, like choosing food for Susan, permissible *only if I do my best to choose well*.^32^ In the restaurant example, choosing well begins and ends at the choosing. But in the case of causing someone to exist, whether the choice was a good one or not depends on my ongoing actions; it depends on what I do to make that chosen existence a good choice once it is underway.

One worry about understanding the moral import of causing existence as an instance of choosing for is that there are many cases in which causing existence does not seem to be a *choice*, in the usual way. Many procreators do not sit down and decide to cause a child to exist. In this sense, causing a child to exist is not usually like choosing a meal.^33^ I assume that there is something of a continuum among progenitors: at one end, there are procreators who sit down and thoughtfully decide whether and when to become parents; at the other, we can imagine a child being created using stolen gametes. It seems clear that the procreators in the former case have chosen existence for the resulting child and are surely makers. It also seems clear that the rightful owners of the stolen gametes in the latter scenario have not—are not even procreators, in any meaningful sense—and should not be understood as makers.

It is likely that there is grey area in the middle, and as such it will not always be clear when we ought to say that someone is the cause of the existence of a child. However, we might take a stab at drawing something of a line (although this line will surely itself admit of vagueness) in pointing out that being the cause of someone's existence must depend on actions, rather than on biological facts. After all, monozygotic twins are very closely genetically related—more so than parent and child—and in some sense one twin springs from the other^34^; yet neither is the maker of the other. On the other hand, procreators who use donor gametes are clearly makers, although they may bear no genetic relation to the resulting child.

The action involved in procreation is not like the action of choosing a dish from a menu. So, one might worry that *choosing* is not the right paradigm at all. However choosing to, say, switch on a lamp is in some sense not usually like choosing: it is like flipping a switch. The sense in which turning on a lamp is choosing to turn on a lamp is the sense of choosing relevant to the moral import of causing existence. If I am under duress, or under hypnosis, and so on, it might not be clear whether my turning a lamp on counts as choosing to turn on a lamp; so, too, with procreation. However, I take it that one need not explicitly *volunteer* in order to be said to have acted so as to choose that another exists; just as one need not explicitly volunteer to turn on a lamp in order to be said to have chosen to turn on a lamp.

One might also worry about the extent to which the decency of an existence is dependent upon social factors, as discussed above. Whether someone has a decent existence or not is heavily dependent upon circumstances out of the control of the chooser. So in that sense, the causal parent cannot singly make it the case that the choice was a good one. But likewise, in the case of the restaurant chooser, whether the meal is good depends heavily on what happens in the kitchen. Still, the restaurant chooser is obliged to do her best insofar as her actions can make the choice good—and so is the causal parent (the maker), because choosing for someone obliges one thusly.^35^

On this account of the moral force of causing existence, the obligation is not rectificatory; it is just what morally permissible choosing for requires. Making it the case that someone exists is only morally permissible if one does one's best to make it the case that that someone exists decently. So, seeing that the child is “content with her condition” is just part of procreating in a morally permissible way.

## 6. Summary

We ought to prefer a causal account of parental obligation—but we must do it in the right way. Causal accounts are strong accounts: on the appropriate conceptualization of causing existence (choosing for), the moral force of causing is clear, easily understood, and generalizable; choosing for generates obligation. However, a simple causal account seems to be tethered to contingent social facts. It also cannot account for the moral role of gamete donors, for biological parents who are not taken to be parents in their “home” societies, for nonhetero-nuclear families, and so on.

On the correct account of parental obligation, I have argued, causing a child to exist generates maker obligation and does so because causing a child to exist is an instance of morally weighty choosing for. Maker obligation is the obligation to do one's best to make the child content with her condition and implies a *pro tanto* obligation to take on the role of parent. Parents, on this account, have parental obligation: the duty to actively care for the child. Parental obligation is fulfilled by adequately parenting the child. Maker obligation is fulfilled by making it the case that the child is adequately parented, either by so parenting or by securing parents for the child. This account, then, retains the strength and plausibility of the simple causal account while dissolving Brake's reasons for rejecting such an account.

## Notes

1 In this context, there is no distinction to be made between “consent” and “volunteer.”

2 See Jeske (1998). See Hardimon (1994) for a critique of this approach.

3 There are, of course, progenitors who are not and do not intend to become parents of their offspring, and we rightly think that the obligations these individuals have to their offspring are different. This will be discussed later in the paper. I will propose that we ought to understand nonparent progenitors as occupying a distinct moral role to that of parents.

4 Who is a parent and who has parental obligation are of course in some sense two ways of asking the same question. So, to insist that unwilling “parents” have parental obligation is just to disagree about who is a parent. However, a voluntarist account can easily assign parental obligation to adoptive parents and does not assign parental obligation to nonparental progenitors. In this sense, it has an advantage over a causal account.

5 Brake (2005, 2010).

6 This is a simplification, in a way. On most causal accounts, parental obligation can be “transferred” via adoption. The worry over “deadbeat” parents is a worry specifically about progenitors who have not so transferred the role of parent and its attendant duties. However, as Brake (2005) points out, this idea of transfer of obligations is problematic, given that we seem only to recognize birth mothers as having transfer rights. Further, it is not clear that obligation transfer even makes sense on a causal account. If causing obliges, it is not clear how or why one could cease to be obliged once one has caused.

7 This is not an insurmountable obstacle for a simple causal account since assigning obligations only to causal parents on causal grounds does not rule out noncausal parents having obligations on other grounds. But it does mean the causal theorist needs to do more work, else her account is incomplete.

8 Fixing parenthood, indeed, might be a worry for *any* role-based account of parental obligation, *including voluntarist accounts*. Since parenthood is cross-culturally slippery, it is not clear why or how we could specify universal entrance conditions. I will set this worry aside for now, although I will note that this may be a worry for my own account of “parental” obligation, but not my account of “maker” obligation (see section 4). Thanks to Stephen Wilkinson for bringing this to my attention.

9 Cf. O'Neill (1979).

10 That is, by having a legitimate claim to parent the child. Since otherwise, I am Prince Charles's mother if I consent to be so.

11 Thomson (1971) writes that “Surely we do not have any such ‘special responsibility’ for a person unless we have assumed it, explicitly or implicitly. If a set of parents do not try to prevent pregnancy, do not obtain an abortion, and then at the time of birth of the child do not put it out for adoption, but rather take it home with them, then they have assumed responsibility for it, they have given it rights, and they cannot now withdraw support from it at the cost of its life because they now find it difficult to go on providing for it. But if they have taken all reasonable precautions against having a child, they do not simply by virtue of their biological relationship to the child who comes into existence have a special responsibility for it” (65).

12 Brake (2010, 171). This broad understanding of consent seems difficult in the face of the strong social pressure people—and especially women—are often under to beget and rear children. Whether we ought to consider nonrefusal under coercion to count as tacit consent is yet another question that I will have to leave to one side.

13 2010, 175. In 2005 (63), Brake argues that unwilling, accidental fathers are responsible for the harm, to the woman, of pregnancy, but not of motherhood. This apparently more restricted claim sits more easily with a voluntarist account, on which motherhood does not follow of necessity from gestation and childbirth.

14 Brake also notes that “intuitions about ‘deadbeat dads’ draw on cultural readiness to blame such men,” and she draws comparison with what she calls “the ‘altruistic stud'—a man who provides sperm to single women who want impregnation” (2010, 175).

15 I take it that the usual way to understand such a situation would be to understand it as both the child and the mother having been harmed, although perhaps we might want to say that the mother is harmed doubly: harmed in being overly burdened (and probably disadvantaged personally) and also harmed insofar as her fulfilling of her duties toward her child has been scuppered.

16 Of course, there is a sense in which we might say that it *is* an intuition about general duty: it seems as though *anyone* in the biological father's position would have such a duty. And that may be right. Anyone who was in the same position to aid as the biological father would have such a duty. But then, anyone who was in the *same* position to aid would *be* the biological father. So, it might be a general obligation in the sense that role obligations are in a way general—it is an obligation that attaches to *anyone in that moral role*.

17 Where “rescue” means something financial, hospital staff may not be in a good position at all to aid. Surely they are not paid enough to support every baby that is born in their hospital. But then, the father may be of limited means himself. We have the intuition that he ought to make sacrifices nonetheless, where we have no such intuition about the nursery nurses who tend to the newborn.

18 Again, Brake's account is a necessity account, rather than a sufficiency account. So, simply taking up the role will not make one a parent without the appropriate entitlement to parent the child. But if this requirement precludes the mother's friends from being the father of the child, then it ought also preclude the tacitly consenting biological father—unless being the biological father is morally salient.

19 One interesting question about role obligations is whether we ought to understand them as special obligations or as general obligations. So, for example, we might want to say that they are general obligations. They are (counterfactually) obligations that everyone has: if the obligation to x is attached to the role R, then it is true for all agents that they are obliged to x iff they occupy the role R.

20 I assume that Mackie's (1965) Insufficient but Necessary member of an Unnecessary but Sufficient set (INUS) condition conceptualization of causation—according to which the A caused B iff A was an insufficient but necessary member of an unnecessary but sufficient set of conditions for bringing about B—is the appropriate model of causation in this context. This model would include birth parents (both genetic and nongenetic), as well as gamete recipients, gamete donors, and in some cases, possibly IVF doctors and technicians; but it would exclude, for example, obstetricians and the parents' matchmakers. I have defended an INUS conceptualization of parental causing in Porter (2012).

21 See also Bayne and Kolers (2003).

22 Procreators who procreate using donated gametes.

23 See Little (1999) for a nice discussion of obligations with respect to moral role entry. One interesting thing to notice about the role of maker is that it seems not to have exit conditions—or rather, it does not seem to be a role one can exit. Parent seems to be a role that one *can* exit—for example, by giving a child up for adoption—even if we might think doing so is morally blameworthy. That makerhood cannot be exited has interesting implications for adoption. See Porter (2012).

24 Archard does not fully defend a causal account of parental obligation, but rather argues that a workable causal account would need to look the way described.

25 Even if, as Brake assumes, our obligations to our children outstrip their rights against us, it is still the case that they have rights against us if and only if we have *at least* corresponding obligations toward them. It is implausible to suppose that we all have, say, some general *duty of parent*, such that children might have a right *against society* to be parented by whoever is in a position to do so. So, if we cannot say that some particular person has an obligation to parent a child, it seems that we cannot say that the child has a right to be parented. For discussion of how children's rights match up with our obligations toward children, see O'Neill (1988).

26 That is, we would not think him blameworthy.

27 The child will also presumably have rights against the social parents (*parents*) if they are different from the causal parents.

28 Benatar (1999) argues that, because gamete donors are so obliged to the resultant child, and because there is no reliable way for gamete donors to ensure that resultant children are adequately parented, most cases of gamete donation are immoral. The claim that there is no reliable way to ensure that the children are adequately parented is an empirical claim that I suspect is false. (Indeed, insofar as it might be true, it is also true that no maker or parent can *ensure* that their child will be adequately parented. Of course, Benatar will not shy away from this claim [cf. Benatar 2008].) While Benatar might be right that gamete donation, as it is often currently practiced, is morally problematic, this does not show that gamete donation is immoral; it shows that donation practices ought to be revised.

29 On the other hand, making explicit the role-bound nature of these obligations has a potentially unintuitive consequence as regards adoption. If maker obligation is a role obligation, and causing a child to exist is what causes one to enter the role, then we are forced to say that maker obligation is nontransferrable since one cannot un-cause a child's existence and thus cannot exit the role of maker. If this is so, then “birth parents” who give their children up for adoption do not thereby transfer their obligation to the child. Like gamete donors, birth parents will not count as *parents* on this account, and so will not have *parental* obligation, since they do not take up the role of parent. But since they continue to be makers of the child even after giving the child up for adoption, they will continue to have maker obligation: the child's well-being will continue to be their (moral) concern. My experience is that intuitions about this claim vary—most people seem to find this claim either totally obvious or totally obviously false. But it seems to sit nicely both with women's actual reasons for seeking abortion (not wanting it to be the case that there is a child in the world who is “theirs,” rather than, say, not wanting to gestate for nine months) and, anecdotally, with birth parents' feelings about the offspring they have given up for adoption. The claim that birth parents are always obliged does not, however, imply that adoption is immoral or illegitimate: other people being obliged to a child does not invalidate the relationship between that child and her parents.

30 *Metaphysics of Morals*, part I, chap. II, section III, title II, §28—p. 99 of Kant (1991).

31 It should be noted that the claim is not that I must do The Best. Rather, it is that I must do *my best*. That is, I must make my best effort to ensure that my choice is a decent one.

32 It has been pointed out to me that one might run into conceptual problems in claiming that makers *choose* for their as-yet nonexistent children since at the time they choose, there is no specifiable person for whom they choose. I do not think this is a worry since we can perfectly well say *after the fact* for whom they chose, and it is only after the fact—that is, only when there is an actual person in existence—that the prior choice becomes morally salient. Analogously, if I plant a bomb on a thirty-year timer, and when it goes off it injures a twenty-year old person, it is still the case that I have bombed the twenty-year-old and am liable to compensate her, and am (probably) blameworthy for having bombed her, and so on. It does not make a difference that she did not exist when I set the timer on the bomb, particularly if we assume that I could have been reasonably certain that *someone* would be a victim of my bomb.

33 Of course, in some sense it almost never is: one cannot choose a particular child to cause the way that one can choose that a particular meal be brought to the table.

34 The metaphysics of twinning is surely out of the scope of this paper, but it does not seem totally incoherent to say that when a zygote/blastocyst divides, one half springs from the other.

35 I am not assuming that only a *good* life can be a good choice. Indeed, I think that a “reasonably content” life, or something of the sort, can be a good choice, much as a reasonably palatable meal can be a good choice for Susan.
